# Metathramycin, a new bioactive aureolic acid discovered by heterologous expression of a metagenome derived biosynthetic pathway†

**DOI:** 10.1039/d0cb00228c

**Published:** 2021-02-02

**Authors:** Luke J. Stevenson, Joe Bracegirdle, Liwei Liu, Abigail V. Sharrock, David F. Ackerley, Robert A. Keyzers, Jeremy G. Owen

**Affiliations:** School of Biological Sciences, Victoria University of Wellington Wellington New Zealand jeremy.owen@vuw.ac.nz; Maurice Wilkins Centre for Molecular Biodiscovery New Zealand; Centre for Biodiscovery, School of Biological Sciences, Victoria University of Wellington Wellington New Zealand; School of Chemical and Physical Sciences, Victoria University of Wellington Wellington New Zealand

## Abstract

Bacterial natural products have been a rich source of bioactive compounds for drug development, and advances in DNA sequencing, informatics and molecular biology have opened new avenues for their discovery. Here, we describe the isolation of an aureolic acid biosynthetic gene cluster from a metagenome library derived from a New Zealand soil sample. Heterologous expression of this pathway in *Streptomyces albus* resulted in the production and isolation of two new aureolic acid compounds, one of which (metathramycin, **6**) possesses potent bioactivity against a human colon carcinoma cell line (HCT-116, IC_50_ = 14.6 nM). As metathramycin was a minor constituent of the fermentation extract, its discovery relied on a combination of approaches including bioactivity guided fractionation, MS/MS characterisation and pathway engineering. This study not only demonstrates the presence of previously uncharacterised aureolic acids in the environment, but also the value of an integrated natural product discovery approach which may be generally applicable to low abundance bioactive metabolites.

## Abstract 2

Heterologous expression of an aromatic polyketide biosynthetic gene cluster recovered from a New Zealand soil metagenome library resulted in the discovery of new bioactive aureolic acids.

## Graphical abstract

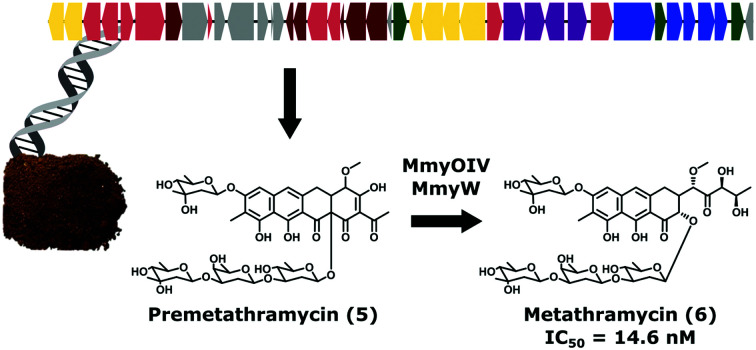

## Introduction

**Fig. 1:**
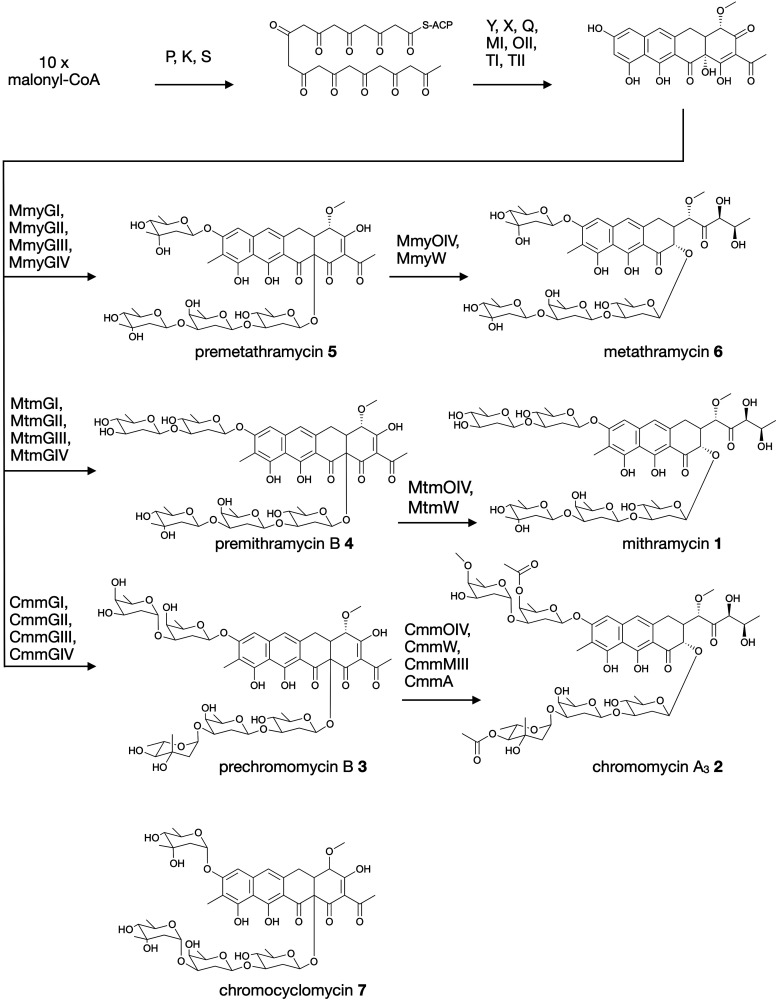
Biosynthesis of aureolic acids mithramycin and chromomycin A_3_, and the structure of chromocyclomycin.

**Fig. 2:**
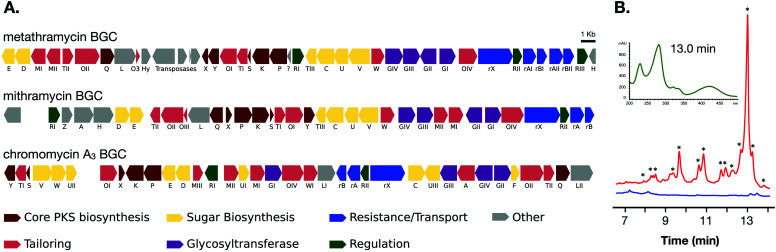
Discovery and heterologous expression of an aureolic acid biosynthetic gene cluster from a soil metagenome library. (A) Comparison of our metagenome derived aureolic acid cluster (MMY cluster) to the known clusters for mithramycin and chromomycin A_3_. Genes are coloured based on broad function classifications. Amino acid identities for alignments of homologous genes are provided in Table 1. (B) HPLC traces for extracts from *S. albus* harbouring the MMY biosynthetic gene cluster (*S. albus*::MMY, red) and an empty vector control (*S. albu*s, blue). Metabolite peaks with the characteristic aureolic acid UV absorbance spectrum (inset, green) are indicated with an asterisk (*). Compound **5** is the major metabolite peak, with a retention time of 13 min. Select minor metabolite peaks were identified by EIC of the LCMS trace; compound **6** is a minor metabolite with retention time 9.4 min, 4-demethylpremithramycinone 7.3 min and premithramycinone (**3**) peak at 9.8 min. The retention time on the reversed-phase C_18_ column is shown on the X-axis, and absorbance at 420 nm on the *Y*-axis.

Aureolic acids are a family of glycosylated aromatic polyketides that have potent bioactivities against human cancer cells and Gram-positive bacteria.^1,2^ All naturally occurring aureolic acids have been discovered from soil or marine actinomycete bacteria, and the biosynthetic gene clusters (BGCs) for two of these have been described: mithramycin (Fig. 1, **1**) from *Streptomyces argillaceus*,^1,3^ and chromomycin A_3_ (Fig. 1, **2**) produced by *Streptomyces griseus* subsp. *griseus.*^4^ Although aureolic acids have been approved for clinical use (*e.g.* mithramycin for testicular carcinoma and myeloid leukemia), their clinical applications remain limited due to significant off target toxicity.^5,6^

**Fig. 3:**
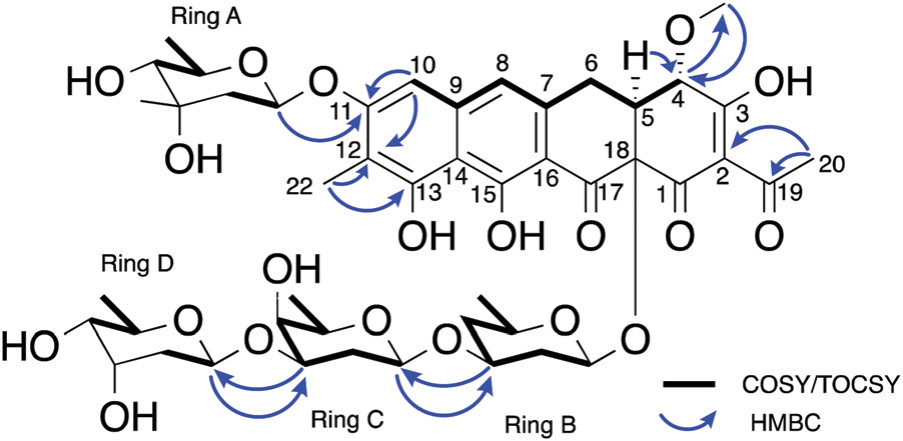
Key NMR correlations used in elucidation of the structure of premetathramcyin: Key HMBC and COSY correlations in the NMR spectra of **5** consistent with the aglycone core of the proposed structure of chromocyclomycin^36,39^ as well as Key HMBC and COSY/TOCSY correlations in the NMR spectra of premetathramcyin providing the glycosidic linkages are shown.

**Fig. 4:**
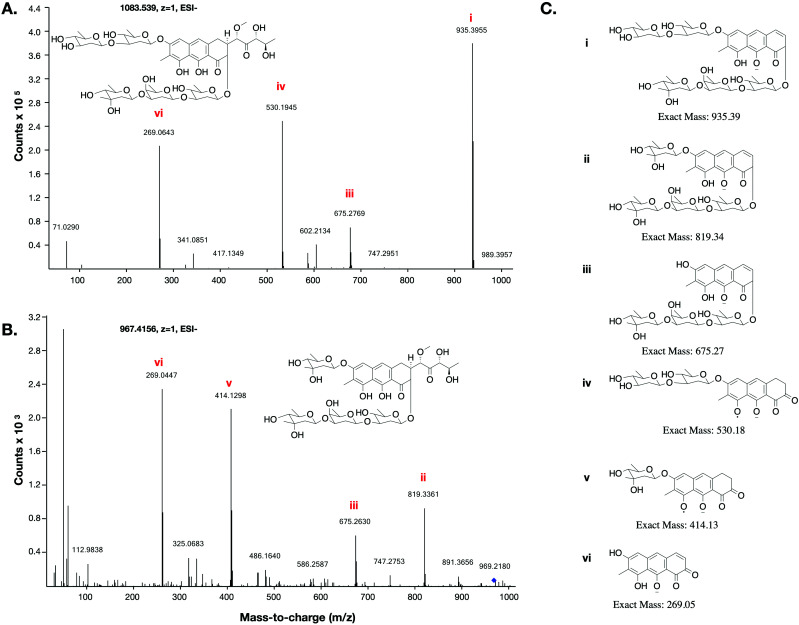
MS/MS fragmentation of mithramycin and metathramycin: MS/MS spectra obtained with a collision energy of 60.0 eV are depicted. (A) Tandem MS showing a possible fragmentation pattern from a sample of mithramycin. Two of the observed daughter ions also occur in panel B. (B) Tandem MS fragmentation pattern of metathramycin. The non-conserved fragment ions *m*/*z* differ from those of mithramycin by 116 (*i.e.* 935–819 and 530–414), equivalent to the mass difference between mithramycin and metathramycin. (C) Putative structures for observed fragment ions.

The potent anticancer bioactivity of mithramycin and chromomycin A_3_ has fuelled a search for new congeners. The search for these mithramycin analogues (“mithralogs”) has predominantly taken the form of gene knockout or combinatorial biosynthesis based on the known biosynthetic genes and pathways for mithramycin and chromomycin A_3_.^6^ There are two main regions of the compound structure that have been targeted for diversity generation. The first is the significant sugar decoration of the polyketide core (premithramycinone, Fig. 1, **3**), which can be modified by deletion or complementation with different sugar biosynthesis pathways and/or glycosyltransferase enzymes Mtm/CmmGI-GIV.^7,8^ The second is the aliphatic tail which is generated by oxidative cleavage of the fourth ring in the tetracyclic precursor (premithramycin B, Fig. 1, **4**) by Mtm/CmmOIV, and subsequent sidechain reduction by Mtm/CmmW. Gene knockout of the sidechain reductase has been demonstrated to produce a set of analogue compounds with distinct toxicity profiles.^2,4^

**Fig. 5:**
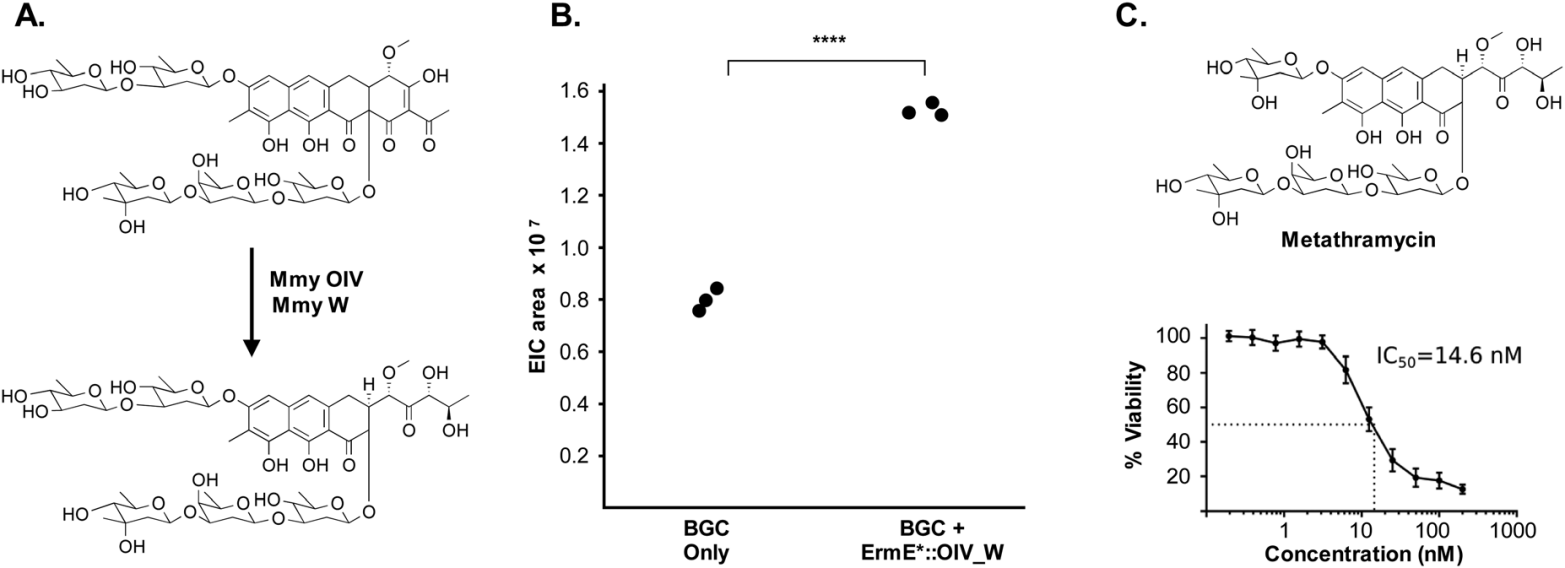
Pathway engineering provides evidence that metathramycin is the product of direct biosynthetic conversion from premetathramycin: (A) reaction catalysed by OIV, W enzymes in the biosynthesis of mithramycin. (B) Overexpression of the OIV, W homologues under the control of a strong constitutive promoter results a 1.9 fold increase in production of metathramycin as compared to a strain containing these genes under the control of a native promoter, data for three biological replicates are shown. (C) MTS assay against the human tumour cell line HCT-116 (*n* = 4) shows metathramycin possesses potent cytotoxicity comparable to that of mithramycin.

Microbial natural products discovered *via* strain isolation and bioactivity guided fractionation have been a phenomenally rich source of medically relevant compounds.^9^ Another method for uncovering novel chemistry from microbes is the discovery of new biosynthetic pathways from environmental microbes. Gene-first natural product discovery by metagenomic analysis has produced numerous novel bioactive secondary metabolites in recent years,^10–15^ providing access to the biosynthetic capacity of microbes independent of our ability to culture these organisms.^16–19^ This approach has proven particularly fruitful when applied to aromatic polyketides, leading to the discovery of improved congeners of clinically relevant compounds.^20^ In this study, we constructed and screened a soil metagenome cosmid library for ketosynthase alpha (KSα) bacterial type II polyketide genes. Sequencing of the recovered library cosmid clones resulted in the partial sequence of an aureolic acid biosynthetic gene cluster with low sequence identity to previously reported clusters. Recovery and reconstitution of the full gene cluster from two overlapping cosmid clones permitted heterologous expression in *Streptomyces albus*, ultimately resulting in the discovery of two new aureolic acid compounds that we have named premetathramycin (Fig. 1, **5**) and metathramycin (Fig. 1, **6**).

## Results and discussion

### Discovery and recovery of novel gene cluster from a soil metagenomic library

Soil collected in Half Moon Bay, Kaikōura, New Zealand, was used as the source of microbial diversity to construct a metagenome library. High molecular weight environmental-DNA (eDNA) was extracted, purified, and cloned into the cosmid vector pWEB::tnc using in-house prepared lambda phage packaging extract.^21,22^ The resulting library of >10 million cosmid clones, each containing 30–40 kb of metagenome DNA, was arrayed over four 96 well plates. These arrayed wells were then screened by PCR with degenerate primers targeting bacterial KSα genes,^23^ using previously established serial dilution plating protocols.^10^

The resulting cosmid clones were pooled and sequenced as a single library (Illumina HiSeq, PE100 bp chemistry). Following assembly with SPAdes,^24^ contigs were matched to individual cosmid insert end sequences by Sanger sequencing, and the assembled metagenome insert sequences analysed with antiSMASH v4.^25^ One of the recovered cosmids had 68% gene cluster similarity by clusterBLAST to the aureolic acid pathway producing mithramycin and 58% to chromomycin A_3_. Notably, however, the individual amino acid sequences encoded by the genes in the partial gene cluster had a relatively low homology to the known aureolic acid biosynthesis pathways, and the gene arrangements were also unique. The recovered cosmid contained only a partial gene cluster sequence, as it was apparent that the *mtrX* gene homologue involved in self resistance and DNA repair was truncated at the cosmid insert boundary.^26^ Other key resistance genes involved in compound efflux in mithramycin and chromomycin A_3_ pathways were not present in the recovered cosmid.^27,28^ Reconstitution of the biosynthetic pathway was achieved by re-screening the soil metagenome library using primers targeting each end of the initially recovered cosmid.^29^ Cosmids extending the recovered sequence in both directions were recovered, however only the sequence that complemented the truncated *mtrX* homologue appeared to contain additional biosynthetic genes. Sequence data for the complete metathramycin BGC has been deposited in GenBank with the accession number MW512268.

The two cosmids containing putative biosynthetic genes were then used to construct a complete and contiguous biosynthetic pathway as a single BAC clone. This was achieved using a modified version of previously published protocols for transformation associated recombination (TAR) in yeast^30^ in which the insert sequences from the two cosmids were liberated from the pWEB::tnc vector using *in vitro* Cas9 digestion with sgRNA targeted to the vector edges. Double-stranded DNA breaks near assembly sites has been demonstrated to increase recombination frequency in TAR,^31^ and the use of targeted Cas9 digestion resulted in free DNA ends within 200 bp of recombination sites. TAR was performed in a previously described *S. cerevisiae* strain deficient in non-homologous end joining^32^ to reduce non-recombinant background colonies, and in a single transformation all emergent colonies (12) analysed tested positive for correct assembly with multiple PCR tests along the BGC length. The reconstituted BGC was then verified by DNA sequencing, and was annotated using results from antiSMASH analysis, as well as BLAST search comparisons of the encoded genes to mithramycin and chromomycin BGCs (Fig. 2A and Table 1). This gene cluster has been assigned the name metathramycin (MMY), as a reference to both the discovery of this gene cluster in a metagenome library, and the gene cluster level similarity to the mithramycin BGC (Table 2).

**Table 1:** Gene cluster comparison of aureolic acid biosynthesis genes: homologous genes between the metathramycin (MMY), mithramycin (MTM) and chromomycin A_3_ (CMM) biosynthetic gene clusters are indicated, with percentage amino acid identities of Mmy proteins to Mtm and Cmy proteins, respectively

Metathramycin BGC	Mithramycin BGC	Chromomycin A_3_ BGC	Proposed function	% aa identity (Mmy:Mtm/Cmm)
		CmmA	O-acyltransferase	
	MtmA		AdoMet synthetase	
MmyC	MtmC	CmmC	NDP-C-methyltransferase	72.7/39.5
MmyD	MtmD	CmmD	NDP-glucose synthase	53.8/53.0
MmyE	MtmE	CmmE	NDP-4,6-dehydratase	63.4/65.8
		CmmF	NDP-5-epimerase	
MmyGI	MtmGI	CmmGI	Glycosyltransferase	57.1/38.9
MmyGII	MtmGII	CmmGII	Glycosyltransferase	58.4/42.2
MmyGIII	MtmGIII	CmmGIII	Glycosyltransferase	66.9/46.0
MmyGIV	MtmGIV	CmmGIV	Glycosyltransferase	67.6/47.7
MmyH	MtmH		Adenosylhomocysteinase	90.1/
MmyHy			Hydrolase	
MmyK	MtmK	CmmK	Ketosynthase β	63.5/64.4
		CmmLI	Acyl-CoA ligase	
MmyL	MtmL	CmmLII	Acyl-CoA ligase	48.3/53.8
MmyMI	MtmMI	CmmMI	O-methyltransferase	56.1/55.1
MmyMII	MtmMII	CmmMII	C-methyltransferase	50.3/54.2
		CmmMIII	O-methyltransferase	
MmyOI	MtmOI	CmmOI	Oxygenase	54.1/56.8
MmyOII	MtmOII	CmmOII	Oxygenase	51.6/53.5
	MtmOIII		Oxygenase	
MmyO3			Oxygenase	
MmyOIV	MtmOIV	CmmOIV	Oxygenase	63.3/57.2
MmyP	MtmP	CmmP	Ketosynthase α	67.6/71.9
MmyQ	MtmQ	CmmQ	Aromatase	50.2/55.9
MmyRI	MtmR	CmmRI	Transcriptional activator	41.7/41.6
MmyRII	MtrY	CmmRII	Regulator	67.4/23.7
MmyRIII			Regulator	
MmyS	MtmS	CmmS	Acyl carrier protein	41.3/49.3
MmyTI	MtmTI	CmmTI	Ketoreductase	50.2/52.3
MmyTII	MtmTII	CmmTII	Ketoreductase	58.5/57.3
MmyTIII	MtmTIII		NDP-4-ketoreductase	63.4/
		CmmUI	NDP-4-ketoreductase	
		CmmUII	NDP-4-ketoreductase	
		CmmUIII	NDP-4-ketoreductase	
MmyV	MtmV	CmmV	NDP-2,3-dehydratase	61.3/46.2
MmyU	MtmU	CmmW	NDP-3-ketoreductase	64.0/52.5
MmyW	MtmW	CmmWI	Ketoreductase	75.1/65.4
MmyX	MtmX	CmmX	Cyclase	52.6/57.0
MmyY	MtmY	CmmY	Cyclase	67.7/70.4
	MtmZ		Thioesterase	
MmrAI	MtrA	CmrA	ATP-binding protein	66.3/52.9
MmrAII			ATP-binding protein	
MmrBI	MtrB	CmrB	Membrane protein	62.4/48.8
MmrBII			Membrane protein	
MmrX	MtrX	CmrX	UV-repair system	63.6/54.0

**Table 2:** Bioactivity assay results of antimicrobial and cytotoxicity assays

	Premetathramycin (**5**)	Tetracycline	Nystatin	Mithramycin A (**1**)	Metathramycin (**6**)
*E. coli* Δ*tolC*	>128	0.125	ND	>32	ND
*B. subtilis* E168	2	1	ND	0.004	ND
*S. cerevisiae* ΔPDR	32	ND	0.5	32	ND
HCT-116	1.91	ND	ND	0.013	0.015

The metathramycin gene cluster has a length of 42 302 bp, similar to mithramycin (42 374 bp) and chromomycin A_3_ (42 074 bp). Of the genes that were assigned to homologous functions, the mean percentage amino acid identity of the products was 60.7% to mithramycin and 52.7% to chromomycin A_3_. In general, the highest amino acid identities in the MIBiG database for most genes in the metathramycin pathway were with genes from the two aureolic acid gene clusters, however this was notably not the case for the first six enzyme functions (core polyketide biosynthesis by P, K, S, and cyclisation/aromatisation by X, Y, and Q). Only Q had its nearest homologue in chromomycin A_3_, while all other genes aligned to other, non aureolic acid type II PKS biosyntheses.

Comparison of the annotated genes within the metathramycin BGC to those of mithramycin^1,3^ and chromomycin A_3_^4^ indicated that all of the genes necessary for biosynthesis of a tetracyclic aureolic acid aglycone premithramycinone (**3**) (*mmyP, K, S, MI, OII, TI, TII*) were present. Also present were genes for the subsequent methylation and glycosylation to a premithramycin B (**4**) analogue (*mmyMII, GI-GIV*) and the final ring opening and sidechain reduction characteristic of aureolic acid biosynthesis (*mmyOIV, W*). There were four glycosyltransferase genes present, indicating at least four sugars are likely to be present in the final product of the biosynthetic pathway. In previously described aureolic acids, the identity, configuration and connectivity of these sugars differed significantly.^1,33–36^ All known aureolic acids contain four to six sugars attached as two oligosaccharide chains composed of combinations of d-olivose, d-oliose d-mycarose and l-chromose B sugars. The metathramycin gene cluster lacked the required genes for chromomycin A_3_ style sugars – the epimerase (CmmF) and subsequent reductase (CmmUIII) enzymes required for the production of l-chromose B, and methyl- and acyltransferase enzymes for further decoration (CmmMIII, A). The lack of a sugar epimerase gene indicates that all glycosylated metabolites of the metagenome pathway expression should only contain d-sugars. The metagenome cluster contained homologues indicative of a full sugar biosynthetic pathway for d-olivose, d-oliose and d-mycarose. The metagenome cluster contains a few notable genes that differ from either of the known aureolic acid biosynthesis pathways: The third oxygenase in the pathway (O3) does not have significant sequence homology to MtmOIII (chromomycin lacks a third oxygenase). The O3 gene has some homology to the DutO3 monoxygenase in dutomycin biosynthesis (51.6% amino acid identity), as the nearest homologue in the MIBiG database. The metagenome gene cluster contains a HAD family hydrolase gene (Hy), which shares low-level identity with a putative hydrolase in streptolydigin biosynthesis (56.4% amino acid identity). Also present is an extra regulatory protein (RIII) that aligns by BLASTp to TetR regulatory proteins.

### Heterologous expression of the biosynthetic gene cluster

The complete BGC was delivered to *S. albus* J1074 by conjugation, and the resulting recombinant strain was cultivated in parallel with an empty vector control in 50 mL R5a medium containing 1 g Diaion HP-20 resin. Following 7 days of cultivation, the resin was collected, eluted with methanol and the resulting crude extracts were analysed by HPLC and LC-MS. Peaks potentially arising from aureolic acids were detected by monitoring absorbance at 420 nm, and these were subsequently confirmed by examining a full absorbance spectrum and comparing this to the characteristic aureolic acid UV signature of a mithramycin standard.^36,37^ The primary findings of these experiments were that the major metabolite of this pathway had an *m*/*z* of 975.3848 [M − H]^−^ (**5**, corresponding to C_48_H_64_O_21_ [M − H]^−^ = 975.3867). However, the HPLC traces had many other smaller peaks with the same distinctive aureolic acid chromophore, some of which corresponded to aureolic acid pathway intermediates (Fig. 2B).

### Isolation and characterisation of premetathramycin

To isolate and characterise the compounds arising from the metathramycin BGC, we scaled up production to a total of 7 L culture in R5a medium, and isolated 7.9 mg of compound **5**, the major metabolic product of the pathway. Compound **5** was a yellow film, and negative mode high-resolution electrospray ionization mass spectrometry [(−)-HRESIMS] analysis detected a deprotonated molecule at *m*/*z* 975.3848, consistent with the molecular formula C_48_H_64_O_21_ requiring 18 double bond equivalents. The UV/vis spectrum of **5** also corroborated the presence of a highly unsaturated and conjugated aromatic compound, with absorbance maxima at 228, 280, 330 and 415 nm consistent with aureolic acid type compounds, while the IR spectrum showed evidence of carbonyl stretches with peaks at 1628 and 1598 cm^−1^ (S12, ESI†). This formula is isomeric with the previously reported aureolic acid compound chromocyclomycin (**7**)^36,38,39^ which is structurally related to mithramycin, however, contains a fourth ring in the aglycone core rather than the tail at C-3 in **1**. Compound **5** had a specific rotation of [*α*]^20^_D_ = −183 (*c* = 0.3, ethanol) comparable to that reported for chromocyclomycin ([*α*]^20^_D_ = −180 (*c* = 0.3, ethanol)).

Although compound **5** was soluble in several individual NMR solvents (CDCl_3_, CD_3_OD, DMSO-d_6_), significant peak broadening was observed and many correlations in the 2D experiments were absent. A mixture of 1 : 1 CDCl_3_ : CD_3_OD gave the sharpest signals, where the ^1^H NMR spectrum showed two aromatic singlet methines at *δ*_H_ 6.85 and 6.71 and numerous other signals indicative of glycosylation, notably four anomeric doublet methines at *δ*_H_ 5.57, 5.37, 4.91 and 4.58 that suggested the metabolite had four sugars. The ^13^C spectrum showed 48 signals, including three highly deshielded carbonyls (*δ*_C_ 200.4, 199.2 and 196.9), 11 sp^2^ hybridised carbons (*δ*_C_ 164.9–101.2), one methoxy (*δ*_C_ 59.8) and eight methyl carbons, four of which (*δ*_C_ 18.4, 18.3, 17.9 and 16.8) correlated (HSQC) with doublet ^1^H resonances in the ^1^H NMR spectrum while the others correlated to singlets (*δ*_C_ 27.4, 26.9, 26.9 and 8.4).

The NMR and UV/vis spectral data was consistent with the aglycone portion of **5** being the same as that reported for chromocyclomycin, which was previously termed chromocyclin.^39^ This structure included the two aromatic singlet methines, of which *δ*_H_ 6.71 showed HMBC correlations to *δ*_C_ 160.4, 112.2 and 108.5 (C-11, C-12 and C-14 respectively) and thus, alongside shared HMBC correlations to C-11 and C-12 with aromatic methyl *δ*_H_ 2.13 (H_3_-22), assigned it to H-10. This proton also showed a ROESY correlation to the other aromatic methine *δ*_H_ 6.85, which placed them on the same side of the aromatic aglycone and was therefore assigned to H-8, as it also showed a weak allylic COSY correlation to the spin system containing H_2_-6, H-5 and H-4 (*δ*_H_ 3.21/2.80, 3.14 and 4.70 respectively). Shared HMBC correlations from H-5 and OCH_3_-21 to C-4 (*δ*_C_ 77.6) placed the methoxy group at C-4, while the acetyl group at C-2 (*δ*_C_ 113.5) was evidenced by HMBC correlations from H_3_-20 (*δ*_H_ 2.49) to C-2 and C-19 (*δ*_C_ 200.4). There were no clear HMBC correlations to connect these two spin systems, however the ^13^C chemical shifts are consistent with other chromocyclin containing congeners premithramycin B and prechromomycin B.^40^ The MS/MS spectrum at low collision energy (20.0 eV) further suggested this tetracyclic structure as opposed to the tricyclic, ring opened structure with a C-3 aliphatic sidechain that is present in many other aureolic acids.^1^ This aliphatic sidechain is the most readily fragmentable section of the metabolite and its loss is clearly observable in the MS/MS at low collision energies for both mithramycin (Fig. S1, ESI†) and metathramycin (Fig. S3, ESI†), however compound **5** shows no such fragmentation (Fig. S2, ESI†). The relative configuration of C-4 and C-5 was determined through interpretation of the ROESY data. Mutual NOE correlations between OCH_3_-21, H-5 and H-6a suggested they were on the same side of the molecule, corroborated by the relatively small coupling constant between H-5 and H-6a (*J* = 4.2 Hz) which indicated a syn relationship. For H-6b, the large coupling constant to H-5 (*J* = 11.0 Hz) and lack of NOE correlations to OCH_3_-21 further supported this assignment. The relative configuration of C-18 could not be determined from this spectroscopic evidence alone.

The carbohydrate moieties of compound **5** were assigned and characterised using a combination of COSY, HMBC, ROESY and HSQC-TOCSY NMR experiments. The anomeric resonance of sugar A, H-A1 (*δ*_H_ 5.57, dd, *J* = 2.2, 9.6), showed an HMBC correlation to C-11 (*δ*_C_ 160.4) thus connecting it to the aglycone core directly, while scalar ^1^H–^1^H coupling constants and ROESY correlations supported a mycarose sugar configuration (Fig. 3). The original reported structure of chromocyclomycin also contained a C-11 mycarose glycoside, present in an α-configuration based on calculations of molecular rotations using Klyne's rule.^36^ The H-*A*1 ^1^H NMR resonance of compound **5** showed a large scalar coupling constant (*J*_*A*1*,A*2_ = 9.6 Hz) consistent with an axial–axial relationship to H_2_-*A*2a (*δ*_H_ 1.57), which suggested the glycoside is β-linked in contrast to that previously reported.

The NMR data of the remaining three carbohydrates bound at C-18 was consistent with an olivose-oliose-mycarose chain, the same as that reported for chromocyclomycin. The mycarose residue was again terminal, and its anomeric centre H-*D*1 (*δ*_H_ 4.91, dd, *J* = 2.1, 9.7) showed an HMBC correlation to oliose C-*C*3 (*δ*_C_ 76.8) while the major scalar coupling constant (*J*_*D*1*,D*2_ = 9.7 Hz) again suggested a β-configuration. The oliose anomeric centre H-*C*1 (*δ*_H_ 4.58) resonance was obscured by the water peak, however the large splitting of H-*C*2a (*δ*_H_ 1.80, q, *J* = 12.2) is consistent with an axial–axial relationship and a β-configuration. Its connection to the olivose residue was determined by the HMBC correlation from H-*C*1 to C-*B*3 (*δ*_C_ 81.0). Although the ^13^C resonance for C-18 was not observed directly, the olivose residue is connected to the aglycone directly based on biosynthetic considerations and is again β-configured based on the large coupling constant to H_2_-*B*2a (*δ*_H_ 1.62, q, 11.7 Hz). The original reports of chromocyclomycin suggested a β-configuration for both olivose and oliose, and an α-configuration for mycarose, thus varying from that determined for compound **5**.

Compound **5** was not found to possess any Gram-negative antibiotic activity when assayed against *E. coli*, consistent with previously characterised aureolic acids. As the test strain lacks the central efflux pump TolC, it is likely that this intrinsic resistance is not mediated by efflux. Compound **5** did possess moderate/strong Gram-positive antibiotic activity (*B. subtilis* E168, MIC 2 μg mL^−1^), however a mithramycin A standard had an MIC of 0.004 μg mL^−1^, indicating 500-fold greater Gram-positive antibacterial potency relative to **5**. Compound **5** also possessed moderate cytotoxic activity against the HCT-116 cancer cell line, with an IC_50_ of 1.91 μM. However, mithramycin A was again far more potent, with an IC_50_ of 0.017 μM.

Chromocyclomycin has only been described in three research publications, where the biological activity is either not defined^36,41^ or it is stated to be “biologically inactive”.^38^ Another analogous compound, the tetracyclic aureolic acid precursor premithramycin B, has also been demonstrated to possess no detectable antibiotic activity (no inhibition of *Micrococcus luteus* growth up to 50 μg in an agar spot bioassay).^40^ However, **5** has here been demonstrated to possess Gram-positive antibiotic activity comparable to that of tetracycline. Likewise, **5** possessed moderate cytotoxicity in the HCT-116 cell line. These results indicate that **5** possesses not only a novel chemical structure, but also a novel bioactivity profile.

### Bioactivity guided fractionation and discovery of metathramycin

The potent bioactivities of aureolic acid family compounds are exclusively found in those with a tricyclic core and aliphatic tail motif (**1**, **2**), which are generated from tetracyclic precursors by characterised ring opening pathway of two enzymes MtmOIV,W/CmmOIV,W.^40,42,43^ The metathramycin BGC contains gene homologues for these enzymes, leading us to speculate that we might have been missing the final product of the pathway. As known mature aureolic acid compounds are highly active Gram-positive antibiotics, we conducted bioactivity-guided fractionation of our fermentation extract to find this putative missing metabolite.

A sample of the crude fermentation extract was fractionated over an HPLC gradient, with fractions testing positive by agar zone of inhibition assays to the *Bacillus subtilis* 168 test strain being pooled and re-fractionated at a shallower gradient. Three rounds of this process culminated in a sample containing an ion with *m*/*z* 967.4186 [M − H]^−^ that possessed potent bioactivity (**6**, corresponding to C_47_H_68_O_21_ calcd 967.4180, *Δ* = 0.6 ppm). Notably, the mass difference between **5** and **6** (976.3928–968.4256 = 7.9672 Da) is the same mass difference between premithramycin B and mithramycin (1092.4414–1084.4727 = 7.9687 Da), indicating a similar biochemical transformation.

The production of **6** in *S. albus*::MMY culture was extremely low, and the amount isolated from 7 L of culture was insufficient to allow use of standard quantification methods. The purified sample was instead quantified by comparison to an injection standard of mithramycin by HPLC and measurements of UV absorption. As mithramycin and the predicted structure of **6** carry identical chromophores, the absorbance of the mithramycin standard was used to quantify the sample of **6**. After normalisation for the mass difference and assuming the extinction coefficient difference is negligible, the calculated quantity of compound **6** was 7.2 μg.

This low yield (∼1 μg L^−1^) precluded structure elucidation by NMR, however MS/MS fragmentation analysis was conducted of **6** and the major biosynthesis product compound **1** against an authentic sample of mithramycin (Fig. 4). Compound **6** fragmented similarly to the mithramycin standard, with some of the fragmented ions shifted by a mass corresponding to the loss of a deoxy sugar and the gain of a methyl group, consistent with the mass difference between mithramycin and **6**. The observed fragmentation pattern in both cases initiated with the loss of the aliphatic sidechain (daughter ion *m*/*z* 820 or 935) at 20.0 eV, then successive loss of deoxy sugars up to 60.0 eV, leaving the tricyclic core at *m*/*z* 269. The observed mass and fragmentation patterns of **6** are consistent with a “ring opened” aureolic acid. This fragmentation pathway was consistent with the gene cluster analysis, where the presence of OIV and W homologues indicate a ring opening step should occur in the biosynthesis pathway as for mithramycin and chromomycin A_3_. Compound **5** did not follow the same fragmentation pattern as the mithramycin standard (Fig. S2, ESI†), potentially due to the lack of an aliphatic tail where the initial fragmentation occurs (at 20.0 eV onwards). Instead, the parent ion of **5** remained intact at 20.0 eV, with only minor fragmentation at 40.0 eV, followed by seemingly disordered breakdown at 60.0 eV, from which three of the conserved core masses observed during fragmentation of both mithramycin and compound **6** could be identified (Fig. S2, ESI†).

The metathramycin BGC contained homologues of both *mtmOIV* and *mtmW*. These are believed to encode enzymes that catalyse oxidative cleavage of the fourth ring in the tetracyclic core of premithramycin, and reduction of the resulting sidechain, respectively, to yield mithramycin (Fig. 5A).^40,43^ To further test the hypothesis that compound **6** was indeed the product of enzymatic opening of the fourth ring of **5**, we amplified the *mmyOIV* and *mmyW* genes from our pathway and cloned these into the integrative vector pIJ10257 as a bi-cistronic unit under control of the constitutive promoter ermE*. The PCR primers were designed to include a unique Shine–Dalgarno sequence^44^ as a point of homology between the two genes to complete the assembly and allow for expression from a single promoter sequence. The resulting construct was integrated into the *S. albus* strain harbouring the complete biosynthetic pathway, and production of **6** quantitated using LC-MS. We found that over-expression of the putative ring opening enzymes resulted in a 1.9-fold increase in **6** production compared to the base expression strain (Fig. 5B), providing further evidence to support the hypothesis that compound **5** is not the (sole) final product of the biosynthesis pathway, and that compound **5** may be converted to compound **6** by the same mechanism as conversion of premithramycin B to mithramycin. Given that **6** appears to be a direct conversion product afforded by ring opening and side chain reduction of **5**, it is reasonable to assume that the configuration and connectivity of sugars is conserved between these two compounds.

Pleasingly, the 7.2 μg that was isolated was sufficient to conduct a bioactivity assay. When tested against the human tumour cell line HCT-116, metathramycin demonstrated potent cytotoxicity comparable to that of mithramycin (Fig. 5C: mithramycin **1** IC_50_ = 13.4 nM, metathramycin **6** IC_50_ = 14.6 nM over 4 biological replicates). Although there was insufficient compound to perform initial toxicology studies, the promising bioactivity of metathramycin against tumour cells suggests that further analysis of this molecule is warranted.

## Concluding remarks

While the task of generating bioactive mithralogs in the laboratory continues, it is apparent that there are aureolic acids in nature yet to be discovered. Metagenome mining studies in recent years have uncovered polyketide biosynthetic pathways that phylogenetically map to aureolic acid biosynthesis.^10^ This study is the first to characterise the aureolic acid chemical output of a metagenome-derived BGC.

Heterologous expression of this biosynthetic gene cluster resulted in a major fermentation product (**5**) that was not the final product of the biosynthetic pathway (**6**). The molecular formula of the major metabolite (**5**) was determined by HR-MS to be C_48_H_64_O_21_, which is identical to a known aureolic acid, chromocyclomycin. Subsequent isolation of **5** revealed that it was not chromocyclomycin, but instead a derivative structure where the glycosidic linkages were exclusively in the β configuration; in contrast, the two mycarose sugars in chromocyclomycin are α-linked. While literature evidence on the bioactivity of chromocyclomycin is limited, our data demonstrated here indicate the different configurations of the mycarose sugars have resulted in a different bioactivity profile between the two compounds.

Uncovering the mature product of the metathramycin gene cluster required us to integrate bioinformatic analysis, pathway engineering, and knowledge from previous biochemical and heterologous expression studies of aureolic acid BGCs. The structure of the major metabolite arising from heterologous expression was not consistent with the gene content of the BGC, suggesting this was not the final product. Bioactivity guided fractionation allowed us to isolate an extremely minor constituent **6** from a complex mixture of biosynthetic intermediates and shunt products, and analysis of fragmentation data from MS/MS experiments allowed a planar structure to be predicted. Finally, overexpression of key genes in the pathway supported our assertion that this was the product of a defined biosynthetic conversion from a metabolite for which we had attained detailed structural data, allowing us to propose likely stereochemistry for the final product of the pathway. Metathramycin was also demonstrated to have potent cytotoxic activity in HCT-116 cells, in keeping with the strong anticancer activities of previously reported aureolic acids. Such a discovery approach may be generalisable to other families of natural product where biosynthetic gene clusters describe well characterised biosynthetic transformations, as has been previously applied for elucidation of stereochemistry.^45^ Without the prior in depth characterisation of aureolic acid biosynthesis by other research groups,^40,42,43^ such an approach would not have been possible, and it is unlikely that the final product of this pathway would have been discovered.

Future studies may now take place with the metathramycin gene cluster as a basis for combinatorial biosynthesis or derivatisation, as has been performed previously in the search for mithralogs.^6^ Other manipulations of biosynthetic pathway regulation may permit greater compound production of **6** to permit further analysis with different cell lines or biological targets.^3,26,28^ The approach used in this study may be applied to future discovery pipelines to find new mithralogs or other low abundance bioactive metabolites.

## Methods

### Construction of soil metagenome cosmid library

A soil sample of ∼1 kg was collected from Half Moon Bay, Kaikōura New Zealand. A sample of 250 g was weighed into a 1 L centrifuge bottle, with 270 mL heated (70 °C) lysis buffer (100 mM Tris–HCl, 100 mM Na EDTA, 1.5 M NaCl, 1% (w/v) cetyl trimethyl ammonium bromide, pH 8.0) added. This was mixed to ensure the soil was thoroughly wetted, and returned to the water bath to reach 70 °C. A volume of 30 mL heated (70 °C) 20% SDS (w/v) was added to the centrifuge tube, inverted to mix, and returned to the water bath. The sample was incubated for 2 h, with gentle inversion every 30 min. The sample was then rapidly cooled in an ice/water bucket for 30 min, with inversion once in this time. The soil and chilled precipitate were then separated from the clarified lysate by centrifugation at 4500 rcf for 35 min at 4 °C. The supernatant was recovered, volume measured, and returned to room temperature by brief incubation in the warm water bath. Isopropanol was added to 0.7 × the supernatant volume, and mixed by gentle inversion to precipitate the eDNA. Following a 30 min room temperature incubation, the precipitated eDNA was collected by centrifugation at 4500 rcf for 35 min at 4 °C. The supernatant was discarded, and the eDNA pellet and centrifuge bottle was washed with 100 mL of ice cold 70% (v/v) ethanol. The sample was again centrifuged at 4500 rcf for 10 min at 4 °C. The supernatant was discarded, and the bottle briefly centrifuged again to collect the remaining ethanol, which was removed by pipette. The eDNA pellet was briefly air dried (no more than 15 min). A minimum volume of TE buffer was added to cover the eDNA pellet, and this was left to slowly resuspend overnight at room temperature.

The eDNA substrate for cosmid library construction was then size selected by agarose gel extraction in 0.8% agarose 1× TAE gel run at 80 V for 2 h alongside λ *Hind*III marker, then overnight in fresh TAE at 18 V. DNA above the 23 kb marker was excised from the gel and electroeluted, then concentrated using a 30 000 kDa molecular weight cut-off column centrifugal concentrator. The DNA sample was quantified by nanodrop, then end-repaired using End-It DNA End-Repair Kit (Epicentre).

The cosmid vector pWEB::tnc was prepared by digested with *Sma*I restriction enzyme (NEB), and dephosphorylation with Antarctic phosphatase (NEB) according to the manufacturer's instructions. The prepared cosmid vector (250 ng) and eDNA substrate (125 ng) were then ligated in a final reaction volume of 5 μL using Fast-link DNA ligase (Epicentre) according to the manufacturer's instructions. Phage packaging extract was prepared in house according to Winn and Norris (2010), and used to package the ligated DNA. The resulting diluted phage heads were added in a 1 : 10 ratio to an ice cold, day culture of *E. coli* EC100 (or EC100 Δ*entD*) grown to OD_600_ of 1.0 in LB 10 mM MgSO_4_. The phage head/cell mixture was incubated at room temperature for 20 min, then aliquoted across 96 wells and incubated at 37 °C 200 rpm for 75 min. The recovered, transfected *E. coli* cells were then diluted in LB Amp Chl for cosmid selection, and titre samples of select wells plates plated on LB Amp Chl agar, for colony counting and library size estimations. The remaining cultures were incubated overnight at 37 °C 200 rpm to replicate the library clones in liquid culture. The following morning, samples were taken from each of the 96 aliquots to make glycerol stocks for long term storage, alongside minipreps of each library “well”.

### Sequencing of clones, bioinformatic interrogation

Cosmids and BACs to be sequenced were isolated from *E. coli* cultures by miniprep, and Macrogen Inc. performed both Sanger and Illumina HiSeq PE100 bp sequencing. Sanger sequencing primers for cosmid end sequencing were standard T7pro and M13F-pUC primers.

Illumina HiSeq (100 bp PE) reads were pre-processed to remove pWEB::tnc vector sequence and contaminating *E. coli* gDNA sequences using bowtie2,^46^ followed by read quality trimming and sequencing adapter removal by Trimmomatic.^47^ The resulting processed Illumina PE reads were then assembled using SPAdes genome assembler.^24^

### Transformation associated recombination

The *E. coli*:yeast:*Streptomyces* shuttle vector, pTARa, was retrofit with a synthetic DNA block *via* Gibson assembly to produce the TAR capture vector. The synthetic DNA contains 712 bp homology capture arms to each of the cosmid insert sequences, separated by a *Pme*I restriction site. To prepare for TAR, the vector was linearised by *Pme*I digestion. Cosmid inserts were liberated from the pWEB::tnc vector by *in vitro* Cas9 digestion. The CRISPR guide RNAs target to the pWEB::tnc vector ∼190 nt upstream of the cosmid insert site (GGTTATTGTCTCATGAG), and ∼115 nt downstream of the cosmid insertion site (GTTAAATTGCTAACGCAGTC).

The three linear DNA units were transformed into *S. cerevisiae* BY4727 *ΔDln4*^32^ for recombination and reconstitution of the BGC with the BAC. The Kallifidias and Brady (2012) protocol was followed for the preparation of yeast spheroplasts, and for the transformation of the spheroplasts with pTARa capture vector and overlapping clones.

### Conjugation into *Streptomyces albus* expression host

Constructs to be conjugated into *Streptomyces* were first transformed by electroporation into *E. coli* S17-1. Overnight cultures of the S17-1 “donor” strain were sub inoculated into fresh LB with appropriate antibiotics, and cultured at 37 °C until OD_600_ reached 0.5–1.0. S17-1 cultures were then chilled and washed in fresh ice-cold LB broth, and resuspended in a final volume of 1 mL LB. The “recipient” *S. albus* J1074 spore stock was diluted into 0.5 mL TSB or LB, and heat shocked at 50 °C for 10 min.

The donor and recipient stocks were then mixed in a 1 : 1 v/v ratio, and centrifuged at 4000 rcf for 5 min. The cell pellet was resuspended in 50–100 μL of the residual LB, and spread onto ISP4 agar, without antibiotics. Plates were incubated for 12–16 h at 30 °C, and then flooded with 1 mL of the appropriate antibiotic stock mix (for pTARa 0.5 mg mL^−1^ nalidixic acid, 1.25 mg mL^−1^ apramycin sulfate, and for pIJ10257 0.5 mg mL^−1^ nalidixic acid, 2.0 mg mL^−1^ hygromycin). Plates were incubated at 30 °C for 24–72 h until colonies emerged.

### Spectroscopic analysis

Optical rotations were measured using a Rudolph Autopol II polarimeter. A JEOL JNM-ECZ600R with a nitrogen cooled 5 mm SuperCOOL cryogenic probe was used to record the NMR spectra of all compounds (600 MHz for ^1^H nuclei and 150 MHz for ^13^C nuclei). The residual solvent peak was used as an internal reference for ^1^H (*δ*_H_ 3.31, CD_3_OD; 7.26, CDCl_3_) and ^13^C (*δ*_C_ 49.0, CD_3_OD; 71.6, CDCl_3_) chemical shifts.^48^ Standard pulse sequences supplied by JEOL were used for NMR analyses. High-resolution (ESI) mass spectrometric data was obtained with an Agilent 6530 Accurate Mass Q-TOF LCMS equipped with a 1260 Infinity binary pump. IR (film) spectra were recorded using a Bruker Platinum Alpha FTIR spectrometer, while UV/vis spectra were extracted from HPLC chromatograms.

Reversed-phase column chromatography was achieved using Supelco Diaion HP20 (PSDVB) chromatographic resin. Size exclusion chromatography was achieved using Sephadex LH20 resin. HPLC purifications were carried out using a Rainin Dynamax SD-200 solvent delivery system with 25 mL pump heads with a Varian Prostar 335 diode array detector. Octadecyl-derivatised silica (C_18_, 5 μm, 100 Å) HPLC columns (Phenomenex) were either analytical (4.6 mm × 250 mm, 1 mL min^−1^) or semi-preparative (10 mm × 250 mm, 4 mL min^−1^), while a Phenomenex Luna octyl-derivatised silica gel (C_8_) column (analytical; 4.6 mm × 250 mm, 1 mL min^−1^) was also used. All solvents used were of HPLC grade and H_2_O was glass distilled. Solvent mixtures are reported as percent volume/volume.

### Production and isolation of premetathramycin and metathramycin

Cultures of *S. albus* J1074::MMY were grown in 7 L total R5a medium, with 1% inoculum from a mature seed culture grown in TSB medium. R5a cultures were prepared with the addition of 20 g L^−1^ of HP20 resin to collect metabolites as they were produced during the 7-day culture time. The HP20 was washed with 1 L of H_2_O, and 1500 mL of 20% methanol/H_2_O, before elution in 1500 mL 80% methanol/H_2_O. The 1500 mL elution was dried under reduced pressure, resuspended in 80 mL of methanol, and undissolved particulate removed *via* centrifugation. The clarified supernatant was dried, with a mass of 1091 mg. Half of this sample was then resuspended in methanol, syringe filtered, and partitioned on an LH20 column run in methanol, with 5 mL fractions collected and combined based on MS analysis for the presence of the protonated molecular ion of **5**. Four early fractions were combined, and were dried under reduced pressure to a dry mass of 262 mg. This sample was purified by semi-preparative C_18_ HPLC with a gradient of 10–100% methanol/H_2_O (0.2% formic acid) over 60 min. Two fractions were collected, one containing the ion for compound **6** (8.0 mg) with a retention time of 51.5 min and another with compound **5** (19.7 mg) that had a retention time of 52.3 min. The first sample was purified by analytical C_18_ HPLC using an isocratic method of 70% methanol/H_2_O (0.2% formic acid), to give compound **6** (7.2 μg). A 10 mg portion of the second sample was also purified by analytical C_18_ HPLC (75% methanol/H_2_O (0.2% formic acid)), to give compound **5** (7.9 mg). This represents a recovery of 4.4 mg L^−1^ of culture (when accounting for the unprocessed sample at each stage of purification).

Premetathramycin (**5**): yellow solid; [*α*]^20^_D_ −183 (*c* 0.3, EtOH); UV (MeOH) *λ*_max_ 228, 280, 330, 415 nm; IR (film) *v*_max_ 3382, 2928, 1628, 1598, 1530, 1370, 1065 cm^−1^; ^1^H NMR (1 : 1 CD_3_OD : CDCl_3_) Tables S1 and S2 ESI;†^13^C NMR (1 : 1 CD_3_OD : CDCl_3_) Tables S1 and S2 ESI;† (−)HRESIMS *m*/*z* 975.3848 [M − H]^−^ (calcd for C_48_H_64_O_21_, 975.3867, *Δ* = −1.9 ppm).

### Antimicrobial testing

Minimum inhibitory concentration (MIC) studies were performed using an established protocol^49^ to test isolated compounds against test strains of *E. coli* Δ*tolC* (7NT)^50^ and *B. subtilis* 168. All tests were carried out in MH medium at 30 °C, with 16 h growth time in biological triplicates (*n* = 3 independent experiments). Positive controls of tetracycline and mithramycin A were included in the analysis. All compounds were assayed from 128 μg mL^−1^ in two-fold serial dilution steps across 20 dilution stages, except for mithramycin, where the starting concentration was 32 μg mL^−1^. Sterility (media only) and growth (cells only) controls were included in each plate assayed.

The same protocol was followed for fungal MICs against *S. cerevisiae* lacking the pleiotropic drug resistance gene PDR4, with the growth medium YPD, and the positive control antibiotic nystatin. The end-point measurement was changed from an eyesight measure to optical density, where a growth inhibited well was one where the OD measurement was <90% of the growth control well (as per EUCAST guidelines for fungal susceptibility testing).

### Tumour cell cytotoxicity assay

A standard 48 h MTS cell proliferation assay was used to evaluate cytotoxic activity against the human colon carcinoma cell line HCT-116 (*n* = 4 independent experiments). Cells were treated with compound at various concentrations, and a dose–response was generated relative to a control of untreated HCT-116 cells. A positive control of mithramycin A was used in all cases.

## Conflicts of interest

There are no conflicts to declare.

## Supplementary Material

CB-002-D0CB00228C-s001
