# Supplementary material for: Metathramycin, a new bioactive aureolic acid discovered by heterologous expression of a metagenome derived biosynthetic pathway
Source: RSC Chem Biol. 2021 Feb 2;2(2):556–67. doi: 10.1039/d0cb00228c (PMC8341913; doi:10.1039/d0cb00228c)

Electronic Supplementary Material (ESI) for RSC Chemical Biology.
This journal is © The Royal Society of Chemistry 2021

Supplementary Material for: Metathramycin, a new bioactive aureolic acid discovered by heterologous expression of a metagenome derived biosynthetic pathway.

Authors: Luke J. Stevenson[1,3,4], Joe Bracegirdle[2,3,4], Liwei Liu[1], Abigail V. Sharrock[1], David F. Ackerley[1,3,4], Robert A. Keyzers[2,3,4] and Jeremy G. Owen[1,3,4 *]

Author affiliations:

1. School of Biological Sciences, Victoria University of Wellington, Wellington, New Zealand
2. School of Chemical and Physical Sciences, Victoria University of Wellington, Wellington, New Zealand
3. Maurice Wilkins Centre for Molecular Biodiscovery, New Zealand
4. Centre for Biodiscovery, School of Biological Sciences, Victoria University of Wellington, Wellington, New Zealand

***Correspondence:** jeremy.owen@vuw.ac.nz

Figures

Figure S1: MS/MS spectra for mithramycin standard……………………………………….….. 2
Figure S2: MS/MS spectra for premetathramycin………………………………………...…. 3
Figure S3: MS/MS spectra for metathramycin………………………………..…….……. 4
Figure S4: ^{1}H spectrum of premetathramycin…………………………………………....... 5
Figure S5: ^{13}C spectrum of premetathramycin………………………………………………… 6
Figure S6: COSY spectrum of premetathramycin…………………………………………..… 7
Figure S7: HSQC spectrum of premetathramycin……………………………….…………… 8
Figure S8: HMBC spectrum of premetathramycin…………………………….……….…..... 9
Figure S9: HSQC-TOCSY spectrum of premetathramycin…………………...……….…..10
Figure S10: ROESY spectrum of premetathramycin…………………………….………... 11
Figure S11: IR spectrum of premetathramycin……………………………….…………...…. 12

Tables

Table S1: Chemical shifts, numbering and correlations for premetathramycin aglycone …… 13
Table S2: Chemical shifts, numbering and correlations for premetathramycin sugars ……… 14

Figure S1 – MS/MS spectra for mithramycin (1) standard ([M-H]$^-$ = 1083.4647):

CID = 20.0 eV

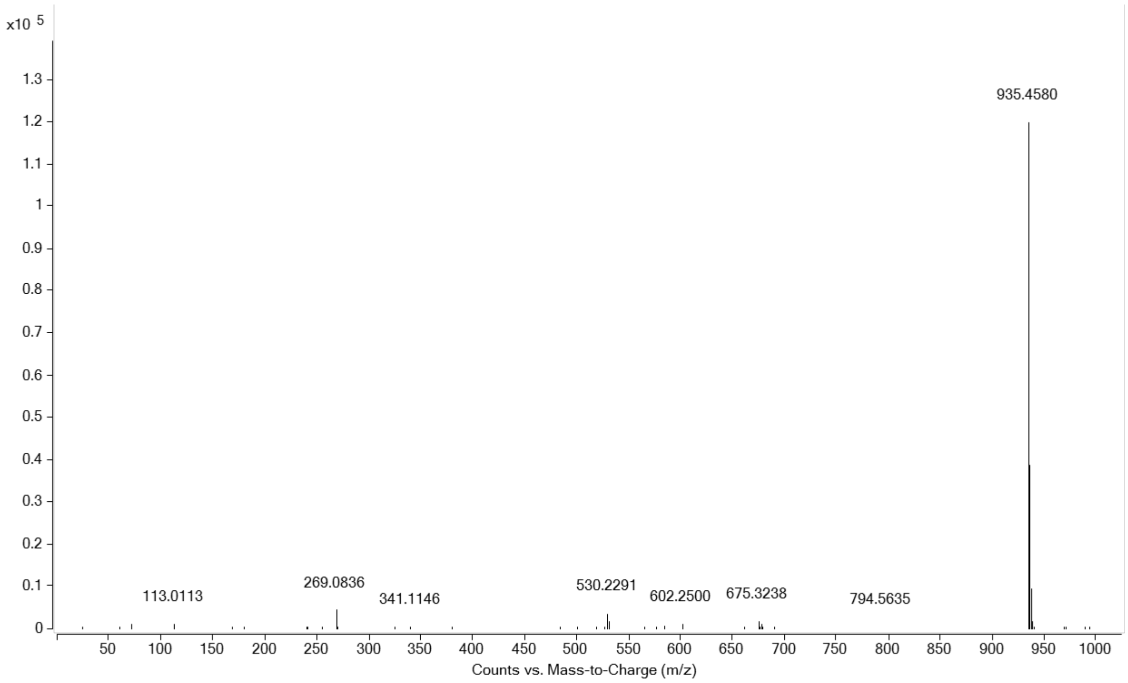

CID = 60.0 eV

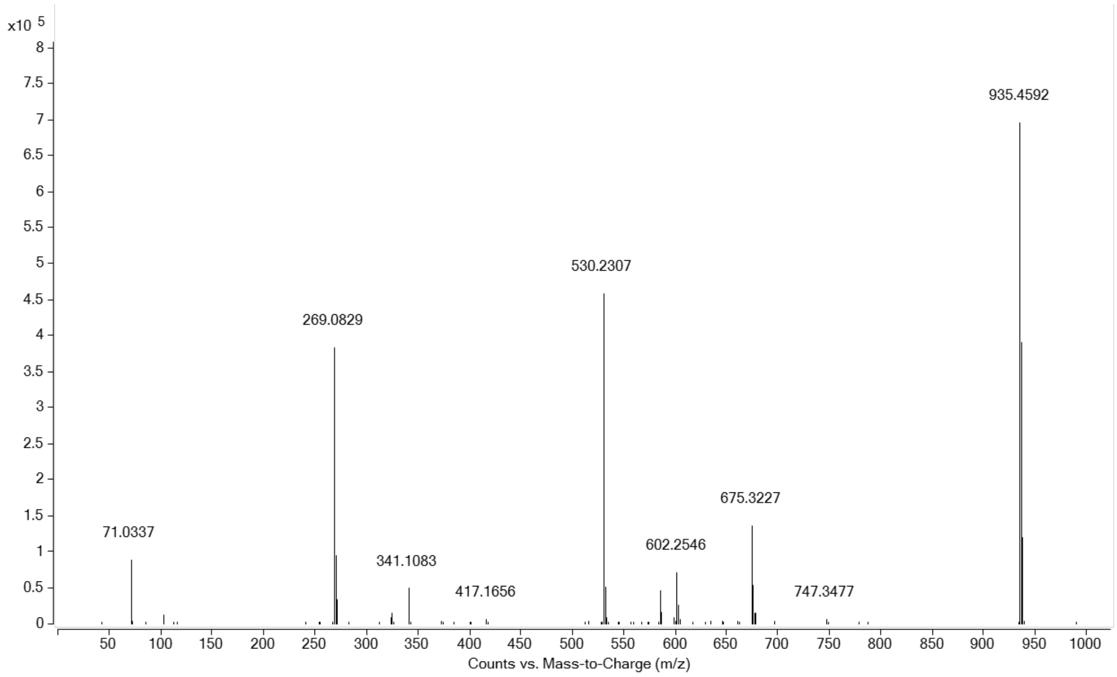

Figure S2 – MS/MS spectra for premetathramycin (5) ([M-H]$^-$ = 975.3848):

CID = 20.0 eV

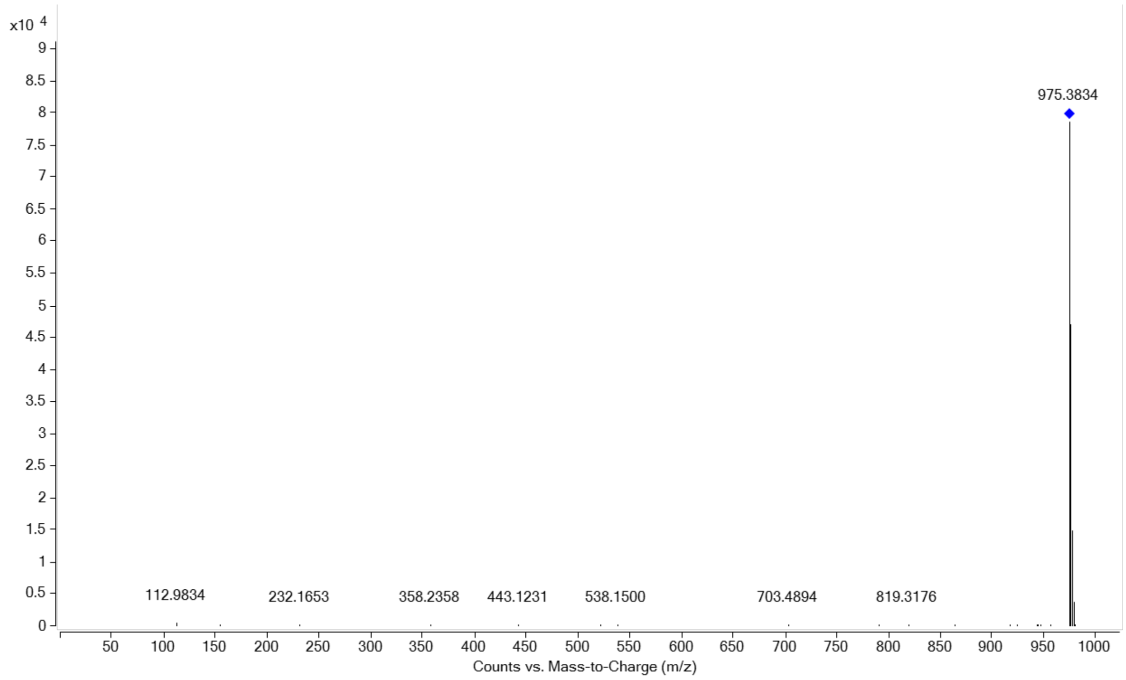

CID = 60.0 eV

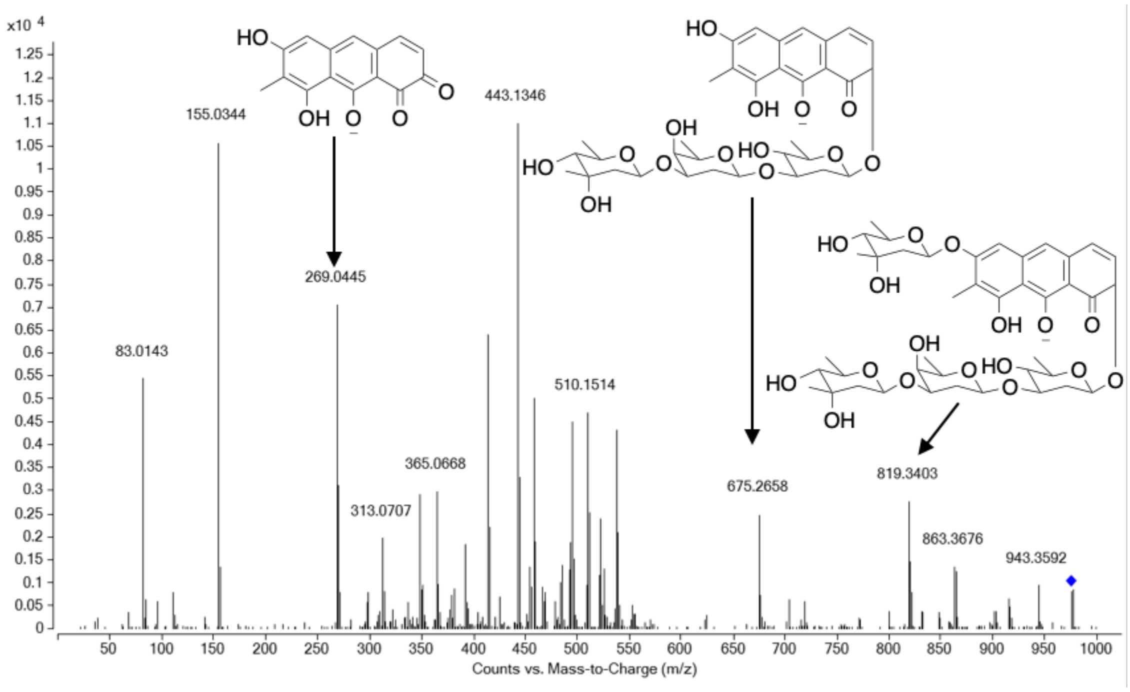

Figure S3 – MS/MS spectra for metathramycin (6) ($[M-H]^-$ = 967.4186):

CID = 20.0 eV

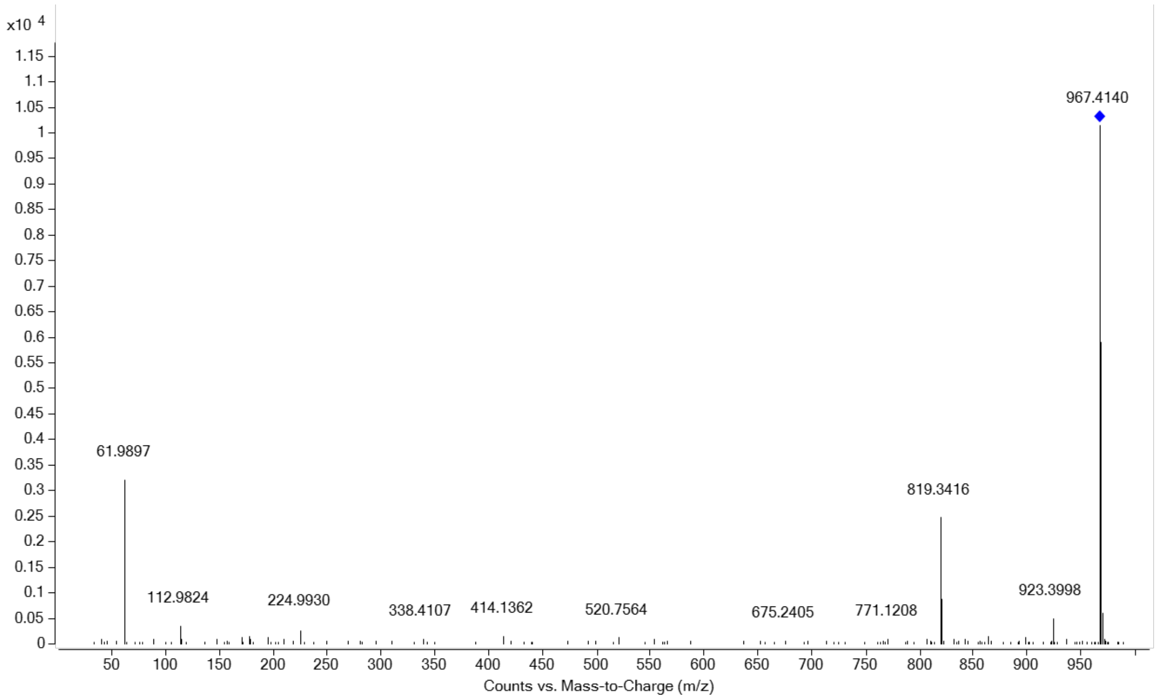

CID = 60.0 eV

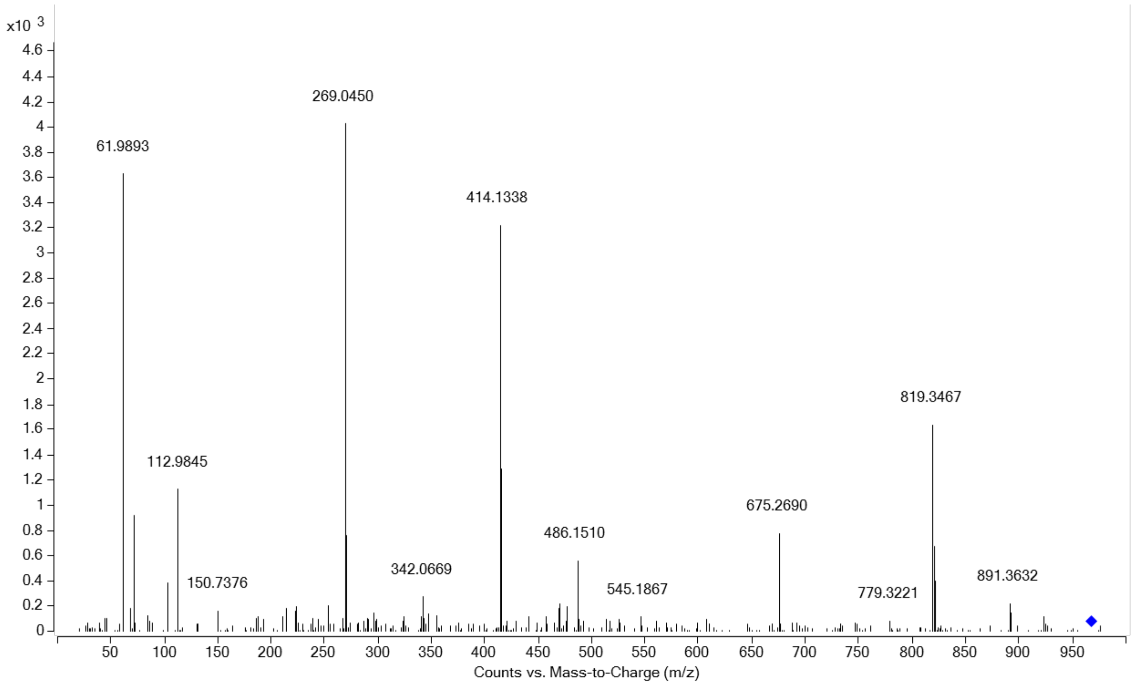

Figure S4 – ^{1}H spectrum of premetathramycin (5) (600 MHz, 1:1 $CDCl_3$: CD_3OD):

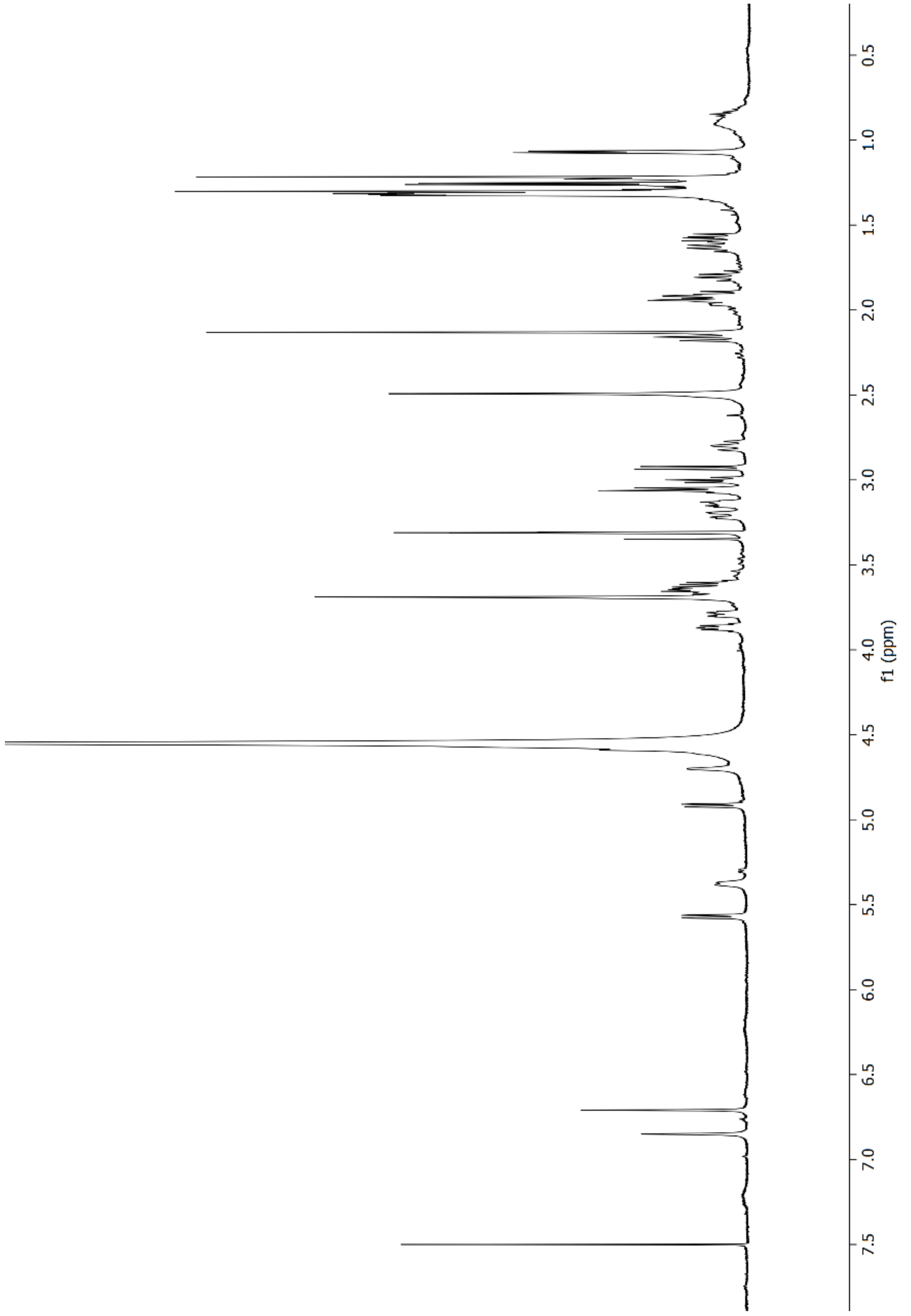

Figure S5 – ^{13}C spectrum of premetathramycin (5) (150 MHz, 1:1 $CDCl_3$: CD_3OD):

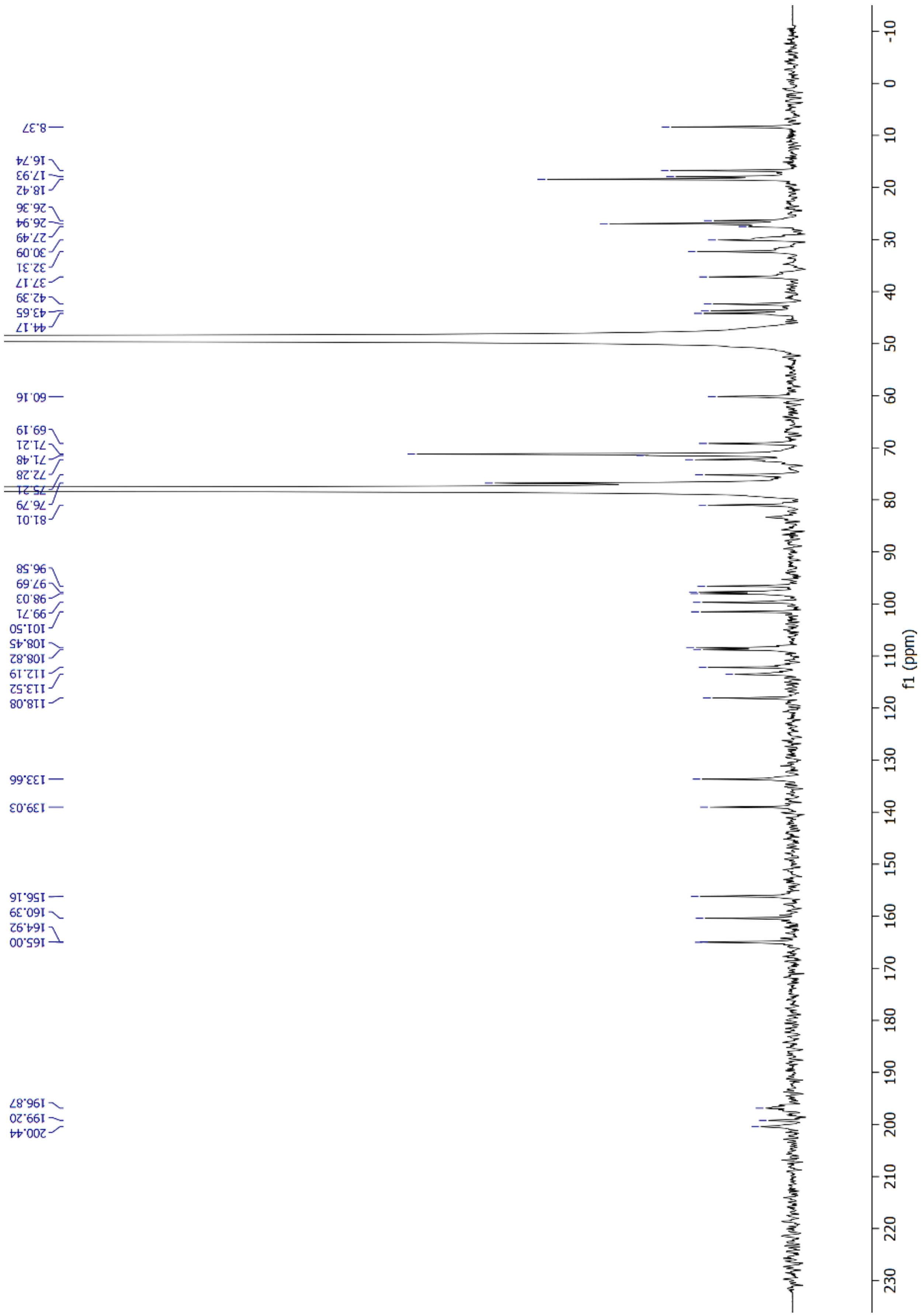

Figure S6 – COSY spectrum of premetathramycin (5) (600 MHz, 1:1 $CDCl_3$: CD_3OD):

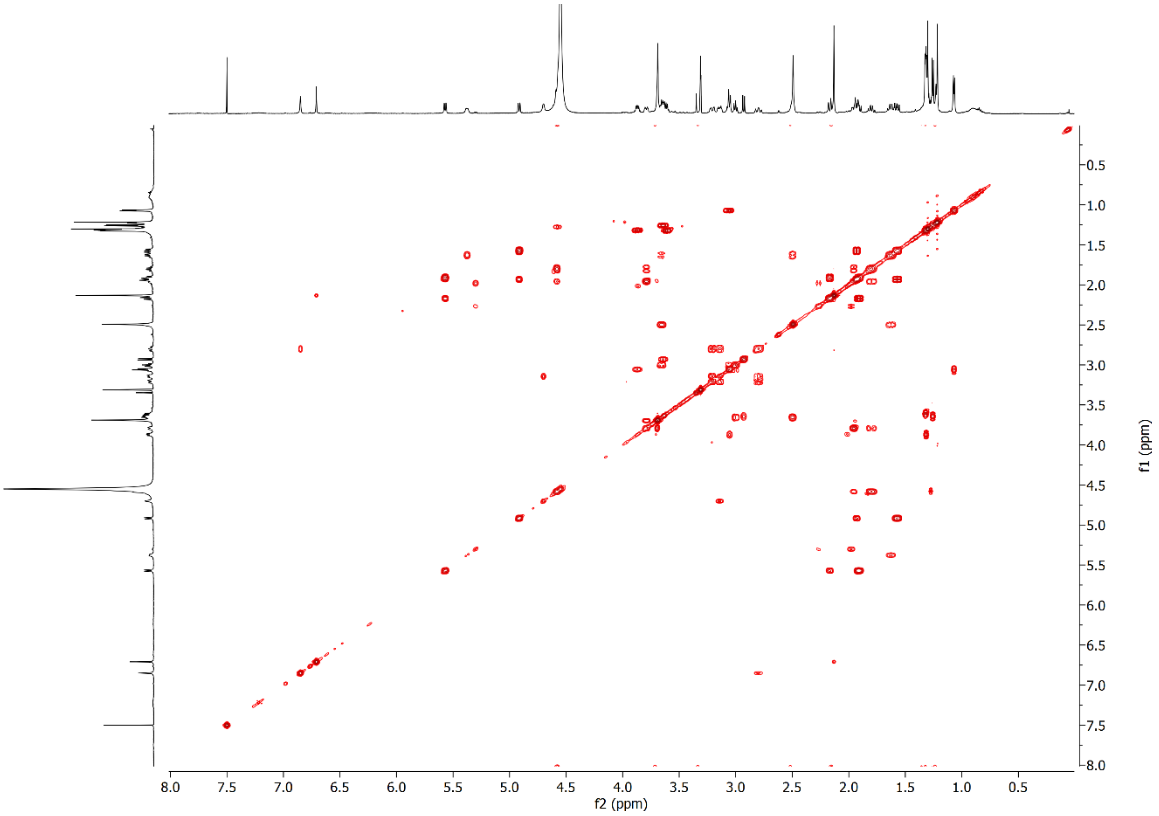

Figure S7 – HSQC spectrum of premetathramycin (5) (600 MHz, 1:1 $CDCl_3$: CD_3OD):

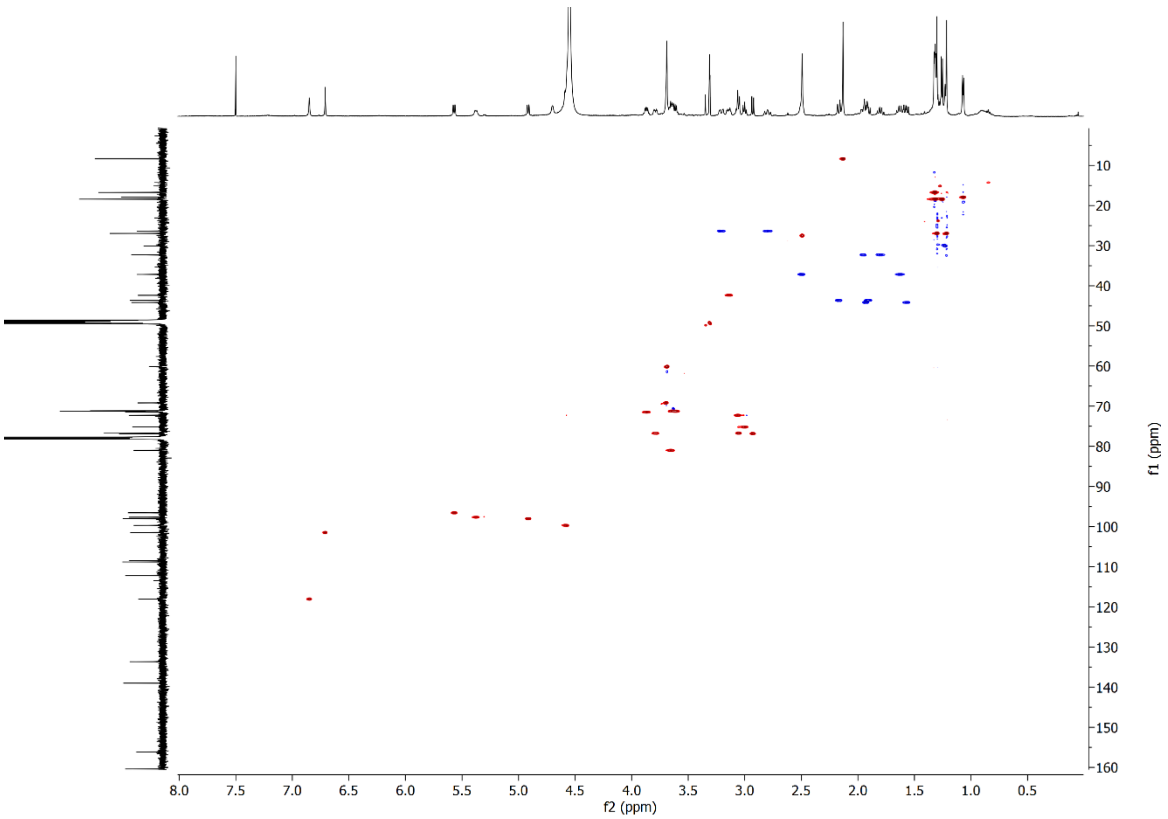

Figure S8 – HMBC spectrum of premetathramycin (5) (600 MHz, 1:1 $CDCl_3$: CD_3OD):

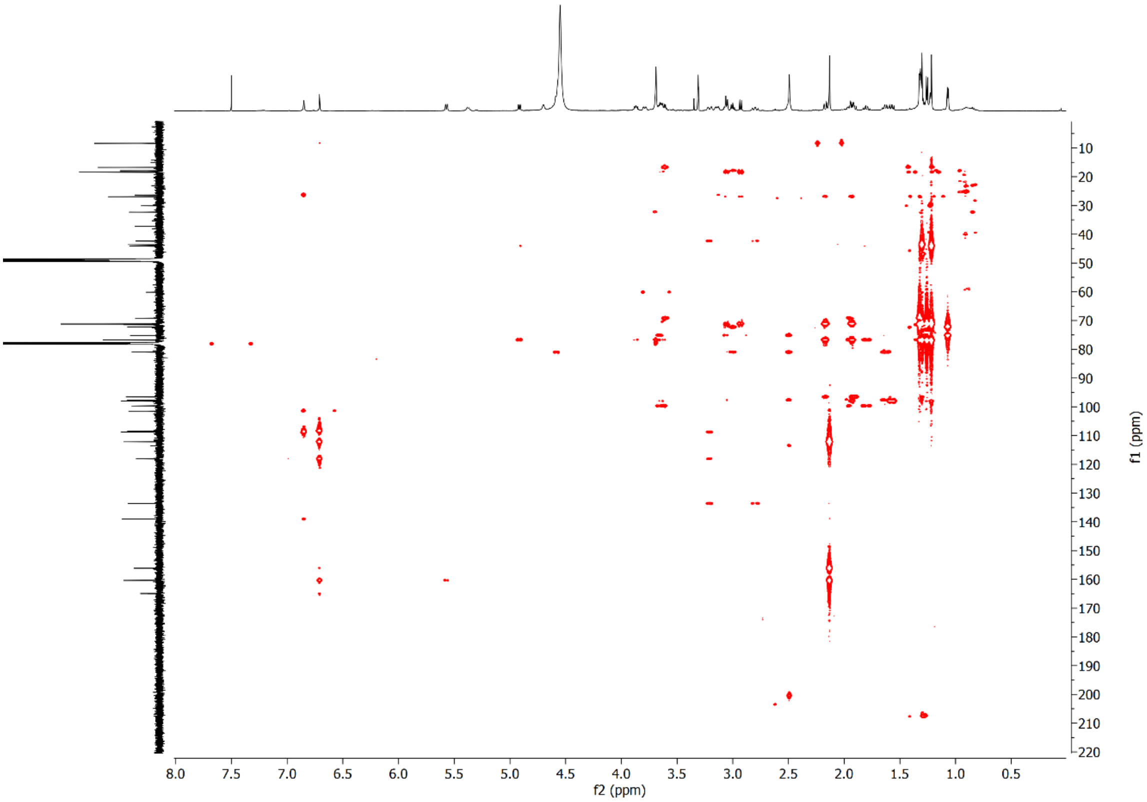

Figure S9 – HSQC-TOCSY spectrum of premetathramycin (5) (600 MHz, 1:1 $CDCl_3$: CD_3OD):

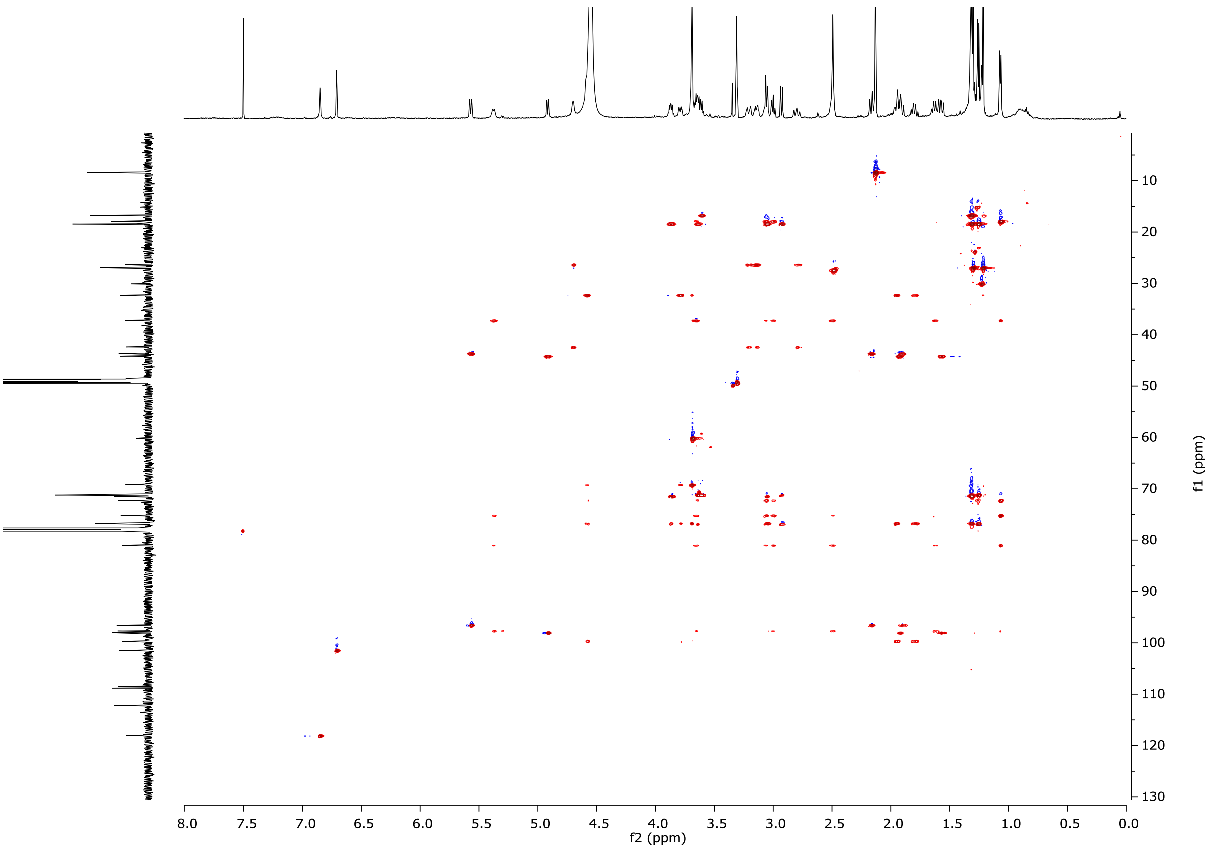

Figure S10 – ROSEY spectrum of premetathramycin (5) (600 MHz, 1:1 $CDCl_3$: CD_3OD):

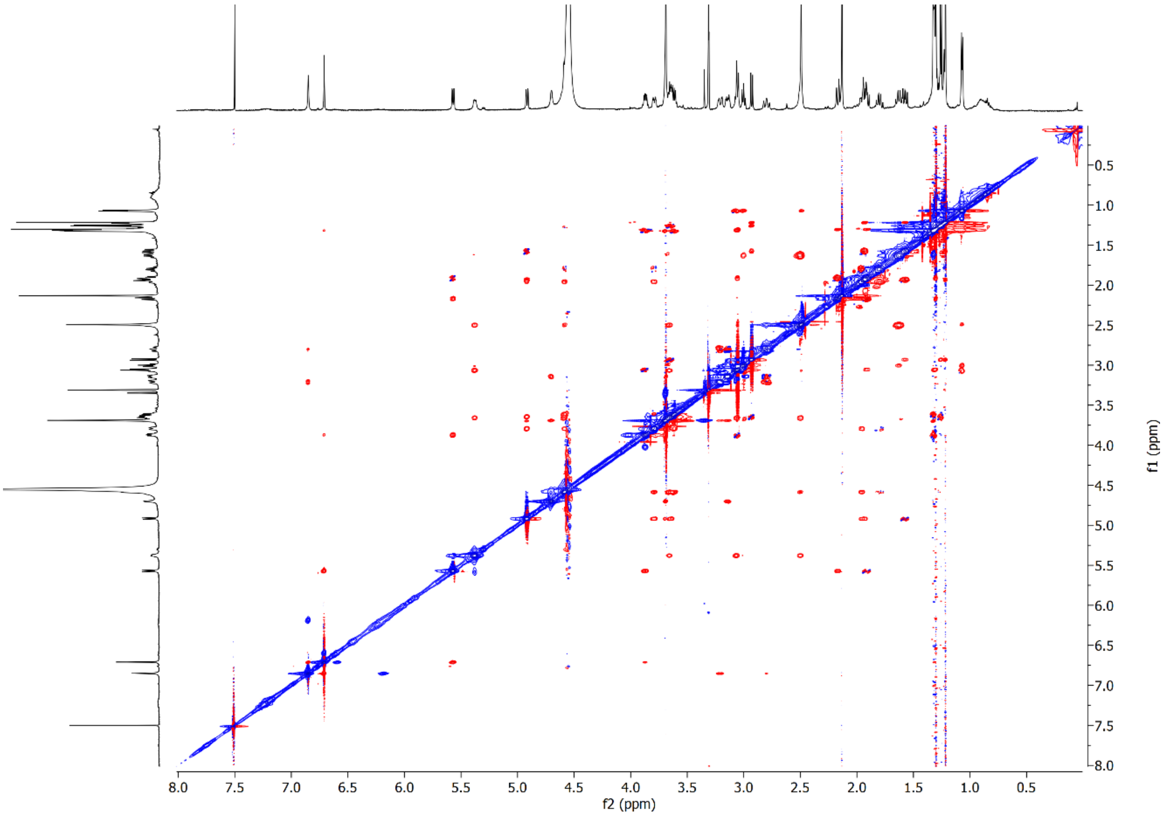

Figure S11 – IR spectrum of premetathramycin (5):

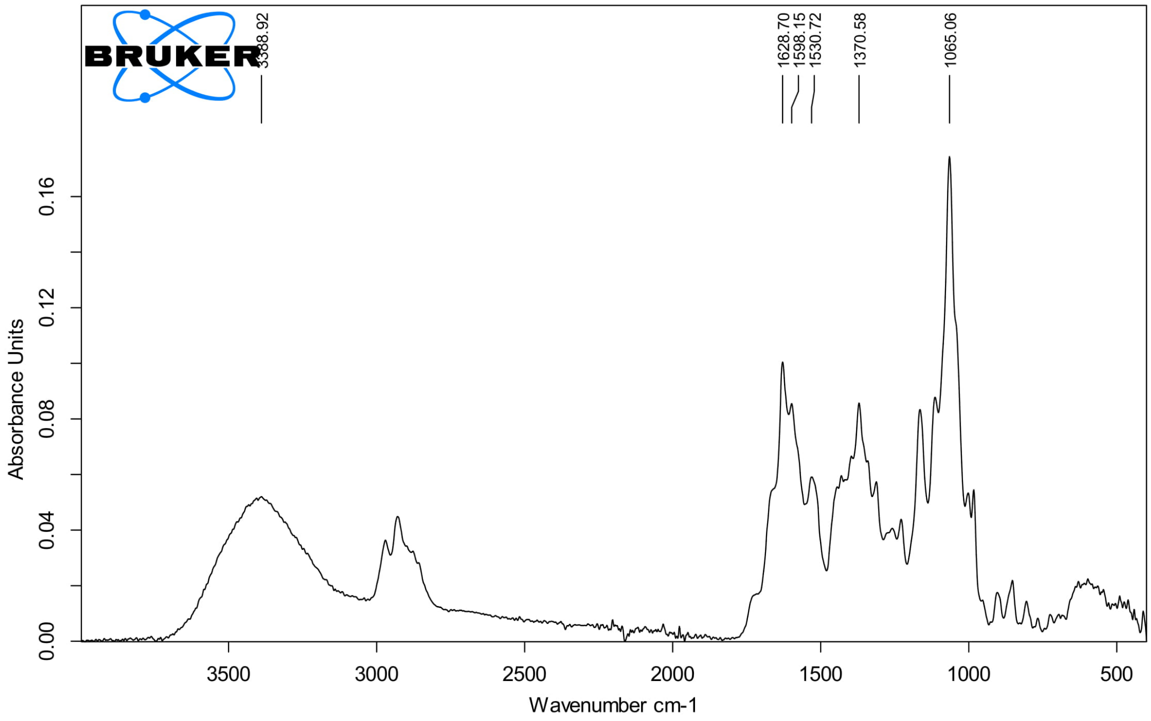

Table S1 - Chemical shifts and correlations for aglycone of Compound 5. ^{1}H (600 MHz) and ^{13}C (150 MHz) NMR data in 1:1 $CDCl_3$:CD_3OD.

position	^{13}C (δ)		^{1}H (δ, mult., *J in Hz)*	COSY	HMBC	ROESY
**1**	196.9	CO				
**2**	113.5	C				
**3**	164.9	C				
**4**	77.6	CH	4.70 (br s)	5		5, 6b, 21
**5**	42.1	CH	3.14 (dt, 10.0, 4.7)	4, 6	6	4, 6a, 6b, 21
**6a**	26.4	CH_2	2.80 (dd, 11.0, 17.0)	5, 8	5, 7	5, 8, 21
**6b**			3.21 (dd, 17.4, 4.2)	5, 8	5, 7	4, 8
**7**	133.5	C				
**8**	117.8	CH	6.85 (s)	6	6, 9, 10, 14, 16	6a, 6b, 10
**9**	139.1	C				
**10**	101.2	CH	6.71 (s)	22	8, 11, 12, 14	8, A1, D6, D7
**11**	160.4	C				
**12**	112.2	C				
**13**	156.2	C				
**14**	108.5	C				
**15**	165	C				
**16**	108.8	C				
**17**	199.2	CO				
**18**	nd	C				
**19**	200.4	CO				
**20**	27.4	CH_3	2.49 (s)		2, 19	
**21**	59.8	OCH_3	3.69 (s)		4	4, 5, 6a
**22**	8.4	CH_3	2.13 (s)	10	11, 12, 13	

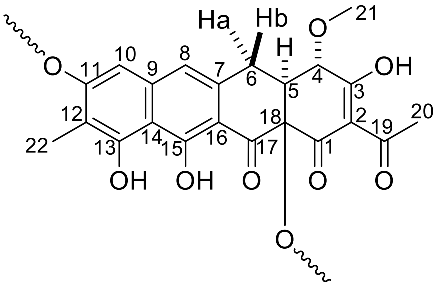

Table S2 - Chemical shifts and correlations for sugars in compound 5 (ol = overlapped). ^{1}H (600 MHz) and ^{13}C (150 MHz) NMR data in 1:1 $CDCl_3$:CD_3OD.

position	^{13}C (δ)		^{1}H (δ, mult., *J in Hz)*	COSY	HMBC	ROESY
**A1**	96.5	CH	5.57 (dd, 9.6, 2.2)	A2	11	10, D6, A2b
**A2a**	43.6	CH_2	1.91 (d, 13.2, 9.6)	A1	A1, A3, A3-Me, A4,	A3-Me
**A2b**			2.17 (dd, 2.2, 13.0)			A1, A3-Me
**A3**	71.2					
**A3-Me**	26.9	CH_3	1.30 (s)		A1, A2, A3, A4,	A2
**A4**	76.8	CH	2.93 (d, 9.4)	A5	A3, A3-Me, A5, A6	A6, D2a, D4
**A5**	71.2	CH	3.64 (ol.)	A4, A6	A4, A6	A6
**A6**	18.4	CH_3	1.26 (d, 6.2)	A5	A4, A5	A4
**B1**	97.6	CH	5.37 (d, 9.7)	B2		B2b, B3, B5,
**B2a**	37.2	CH_2	1.62 (q, 11.7)	B1, B3	B1, B3, B4	B4
**B2b**			2.50 (ol.)		B1, B3, B4	B1
**B3**	81.0	CH	3.66 (ol.)	B2, B4	B4, C1	B1
**B4**	75.5	CH	3.01 (t, 8.8)	B3, B5	B3, B5, B6	B2a, B6
**B5**	72.2	CH	3.05 (ol.)	B4, B6	B1, B4, B6	B1
**B6**	17.9	CH_3	1.07 (d, 6.0)	B5	B4, B5	B4
**C1**	99.7	CH	4.58 (ol.)	C2	B3, C2	C2b, C3, C5
**C2a**	32.3	CH_2	1.80 (q, 12.0)	C1, C3	C1, C3, C4	
**C2b**			1.95 (ol.)		C1, C3, C4	C1, C3
**C3**	76.8	CH	3.79 (dt, 12.0, 4.0)	C2, C4	D1	C1, C4, C5, D1
**C4**	69.1	CH	3.69 (ol.)	C3, C5	C2, C3	D1, C5
**C5**	71.1	CH	3.61 (ol.)	C4, C6	C1, C3, C4, C6	C1, C3, C4
**C6**	16.8	CH_3	1.32 (d, 6.7)	C5	C3, C4, C5	
**D1**	98	CH	4.91 (dd, 9.7, 2.1)	D2	C3, D2	C3, C4, D2b
**D2a**	44.2	CH_2	1.57 (dd, 13.8, 9.7)	D1	D1	A4, D3-Me
**D2b**			1.93 (ol.)		D1, D3, D3-Me, D4	D1, D3-Me
**D3**	76.7	C				
**D3-Me**	26.9	CH_3	1.22 (s)		D1, D2, D3, D4	D2a, D2b
**D4**	76.8	CH	3.05 (ol.)	D5	D3, D3-Me, D5, D6	A4, D6
**D5**	71.5	CH	3.87 (dq, 8.3, 6.0)	D4, D6	D3, D4, D6	
**D6**	18.3	CH_3	1.31 (d, 5.8)	D5	D4, D5	A1, D4

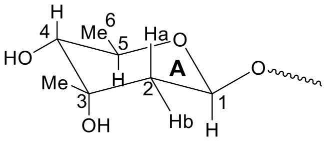

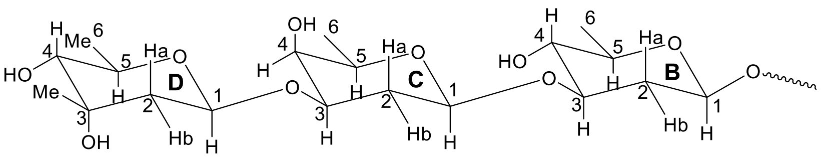

Supplement: CB-002-D0CB00228C-s001 [file CB-002-D0CB00228C-s001.pdf]
